# Transcriptome profiling of the dynamic life cycle of the scypohozoan jellyfish *Aurelia aurita*

**DOI:** 10.1186/s12864-015-1320-z

**Published:** 2015-02-14

**Authors:** Vera Brekhman, Assaf Malik, Brian Haas, Noa Sher, Tamar Lotan

**Affiliations:** Marine Biology Department, The Leon H. Charney School of Marine Sciences, University of Haifa, 31905 Haifa, Israel; Bioinformatics Service Unit, University of Haifa, 31905 Haifa, Israel; Broad Institute of Massachusetts, Institute of Technology and Harvard, Cambridge, Massachusetts USA

**Keywords:** *Aurelia aurita*, Jellyfish, Scyphozoa, Transcriptomics, Life-cycle stages

## Abstract

**Background:**

The moon jellyfish *Aurelia aurita* is a widespread scyphozoan species that forms large seasonal blooms. Here we provide the first comprehensive view of the entire complex life of the *Aurelia* Red Sea strain by employing transcriptomic profiling of each stage from planula to mature medusa.

**Results:**

A de novo transcriptome was assembled from Illumina RNA-Seq data generated from six stages throughout the *Aurelia* life cycle. Transcript expression profiling yielded clusters of annotated transcripts with functions related to each specific life-cycle stage. Free-swimming planulae were found highly enriched for functions related to cilia and microtubules, and the drastic morphogenetic process undergone by the planula while establishing the future body of the polyp may be mediated by specifically expressed Wnt ligands. Specific transcripts related to sensory functions were found in the strobila and the ephyra, whereas extracellular matrix functions were enriched in the medusa due to high expression of transcripts such as collagen, fibrillin and laminin, presumably involved in mesoglea development. The *CL390-*like gene, suggested to act as a strobilation hormone, was also highly expressed in the advanced strobila of the Red Sea species, and in the medusa stage we identified betaine-homocysteine methyltransferase, an enzyme that may play an important part in maintaining equilibrium of the medusa’s bell. Finally, we identified the transcription factors participating in the *Aurelia* life-cycle and found that 70% of these 487 identified transcription factors were expressed in a developmental-stage-specific manner.

**Conclusions:**

This study provides the first scyphozoan transcriptome covering the entire developmental trajectory of the life cycle of *Aurelia*. It highlights the importance of numerous stage-specific transcription factors in driving morphological and functional changes throughout this complex metamorphosis, and is expected to be a valuable resource to the community.

**Electronic supplementary material:**

The online version of this article (doi:10.1186/s12864-015-1320-z) contains supplementary material, which is available to authorized users.

## Background

Cnidarians, such as coral, sea anemone, jellyfish and hydra, are distributed worldwide and play important roles in shaping marine ecosystems. They are dated back to about 700 million years and are considered a sister group to the Bilateria [1,2]. Being diploblastic organisms, with two germ layers of ectoderm and endoderm separated by an extracellular matrix (ECM) of mesoglea, they are among the simplest animals at the tissue level of organization. In addition to their morphological simplicity they have a high level of developmental plasticity that equips them for shape transformation, regeneration and asexual proliferation during their life cycle. These unique characteristics, together with their basal position in the evolutionary tree, make the cnidarians an important group for studies leading to a deeper understanding of basic developmental and evolutionary processes.

The jellyfish, in which the medusa phase is the dominant part of the life cycle, comprise the smallest cnidarian group, containing fewer than 250 species that together represent about 2−3% of the Cnidaria phylum [3]. The most prominent of the jellyfish groups are the Scyphozoa, whose periodic large blooms often have destructive effects on marine biodiversity, fishery and industry [3-5]. These outbreaks results from natural environmental fluctuations combined with the effects of xenobiotic agents and lead to rapid asexual proliferation [6-11].

**Figure 1:**
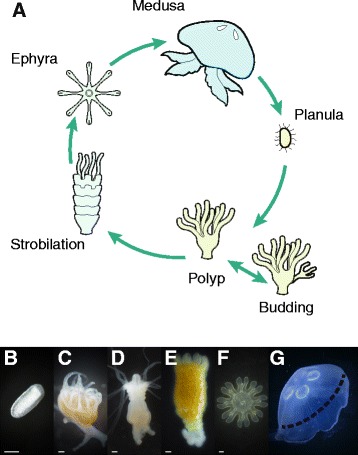
**Life-cycle stages of Aurelia. (A)** Life-cycle scheme depicting sexual reproduction of mature medusa and asexual proliferation of the polyp. **(B − G)** Photographs of the six analysed stages: planula **(B)**, polyp **(C)**, early strobila **(D)**, advanced strobila **(E)**, ephyra **(F)** and mature medusa **(G)**. The dashed line in G represents the excised part of the medusa that was used for RNA-seq. Bar in B, 50 μm and in C − F, 500 μm.

The moon jellyfish, *Aurelia aurita*, is a cosmopolitan scyphozoan species that habituates a wide range of temperatures and has been subjected to diverse ecological and molecular studies [9,12-15]. Like most jellyfish, *Aurelia* has a complex life cycle that typically incorporates both sexual and asexual proliferation, with sexual reproduction occurring at the medusa stage and polyps serving as the main form for asexual reproduction (Figure 1). During embryogenesis a swimming planula (larva) emerges, and after settlement differentiates by metamorphosing into a mature sessile polyp. Polyps, the most stable form of the jellyfish life cycle, can reproduce asexually by budding to produce large polyp cultures. Transition from the polyp stage to the medusa stage occurs through strobilation, an orderly developmental process of metamorphosis in which transverse constrictions subdivide the polyp body from the oral to the aboral end into segmental discs. Each segment develops into a complete young medusa, called an ephyra, which is sequentially liberated from the polyp predecessor (Figure 1). The remaining sessile aboral stump regenerates into a new polyp which, under the appropriate induction, can again undergo transformation to the strobilation phase. The free-swimming ephyra grows into a mature medusa that can reproduce sexually.

To understand the molecular process underlying jellyfish proliferation, different laboratory experiments were performed demonstrating that stressful conditions such as temperature change or the presence of iodine and indole derivatives can trigger the strobilation process [8,13,16-18]. A recent transcriptomic study by Fuchs et al. [13] characterizing three stages in the *Aurelia* life cycle, resulted in the discovery of part of the hitherto unknown molecular cascade of strobilation, revealing the importance of retinoic acid signalling and identifying novel *Aurelia* peptides that may act as strobilation hormones.

Here we applied a transcriptome-wide approach to study the complete life cycle of the *Aurelia aurita* Red Sea (RS) strain, which contains six developmental stages from the emerging planula to the mature medusa. We identified stage-specific gene expression profiles that distinguish unique bioprocesses characterizing each *Aurelia* life-cycle stage. In addition, we identified candidate genes with possible key roles at each developmental stage.

## Results

### De novo transcriptome assembly of *Aurelia* life-cycle stages

Fertile *Aurelia* medusae were collected during the spring of 2012 from the Red Sea in Eilat, Israel, and planulae were isolated for generation of polyp culture stocks. The polyps routinely reproduced asexually at 18°C, and strobilation occurred when the temperature was increased to 25°C. Phylogenetic analysis of the collected *Aurelia* samples demonstrated close relationships with other samples that were previously collected from Eilat [19] (Additional file 1). To study the underlying molecular mechanisms during the *Aurelia* life cycle we carried out transcriptomic profiling of six stages (Figure 1) comprising: (1) planulae isolated from mature *Aurelia* medusae; (2) polyps ; (3) early strobilae demonstrating initial transverse constrictions; (4) advanced strobilae with three or more developed segments; (5) ephyrae, 1–3 days old; and (6) medusae samples with a bell diameter of about 30 cm, of which only the central portion of the bell incorporating exumbrellar and subumbrellar epithelial and gonadal tissues was excised for analysis, excluding the bell margins containing the medusa rhopalia sensory structures and the marginal tentacles. At each stage we extracted RNA and subjected it to sequencing using an Illumina HiSeq-2000 sequencing machine. This generated a total of 162 million pairs of 100-base-length paired-end reads, with an average of 27 (±4.9) million paired-end reads over the six life-cycle stages.

Using the Trinity software, we assembled the pooled reads into a transcriptome [20,21], as no reference genome is yet available for *Aurelia*. The de-novo assembly had a total length of about 180 Mb and resulted, after removal of contaminating sequences (see Methods), in 252,170 transcript isoforms and 131,464 unique transcripts, with an N50 of 1222 and GC percent of 39.52% (Table 1). To assess the quality of the assembly we aligned the input reads to the transcriptome from each stage separately. On average, 80% of the reads mapped back to the assembly, indicating that the assembly represented the majority of sequenced reads. The completeness of the transcriptome assembly was assessed using the CEGMA subset of 248 widely conserved eukaryotic core genes that are considered to have low frequencies of gene family expansion [22,23]. The analysis indicated that 92% of the core genes were completely assembled and an additional 3% were partially assembled resulting in a total of 95% representation of the core genes in the transcriptome (Additional file 2). The transcriptome contiguity [21,24] was tested against the *Hydra magnipapillata* NCBI protein database, as this is the closest organism with a published genome. The analysis indicated that 61% of the matching database proteins are covered by over 70% of their length by the longest matching transcriptome contig (Additional file 3). The large number of fully reconstructed transcripts supports the high quality and contiguity of the assembled transcriptome.

**Table 1:** **Assembly statistics**

	**Total**	**Reduced (FPKM ≥1.5)**
Number of transcripts	252,170	46,657
Number of unique transcripts	131,464	24,264
Total length of transcripts (bp)	180,199,034	53,022,565
Mean transcript length (bp)	715	1136
Median transcript length (bp)	371	727
N50 (bp)	1,222	1,761
GC %	39.52	39.68

As we were less interested in the low-abundance transcripts, we generated a subset consisting only of transcripts with a minimum FPKM (fragments per kilobase per million fragments mapped) of 1.5 in at least one developmental time point. This reduced set of transcripts yielded 46,657 transcript isoforms, 24,264 unique transcripts and a higher N50 of 1761 (Table 1). While such filtering may have biased our data set towards more highly expressed genes, it also yielded a more contiguous assembly, as the percentages of sequences larger than 1000 bp were doubled and the percentages of the short sequences were reduced by half (Table 2). This was expected, as transcripts that are more highly expressed have more reads representing them and thus have a better chance of being assembled contiguously.

**Table 2:** **Summary of transcript length distribution in the de novo transcriptome**

**Length**	**Total**		**Reduced**	
**(bp)**	**Transcripts**	**%**	**Transcripts**	**%**
200-500	158,767	62.96	15,994	34.27
501-1000	46,245	18.34	12,898	27.64
1001-1500	18,082	7.17	6,566	14.07
1501-2000	10,340	4.10	4,064	8.71
2001-2500	6,712	2.66	2,638	5.65
2501-3000	4,282	1.70	1,643	3.52
>3000	7,742	3.07	2,854	6.11

**Figure 2:**
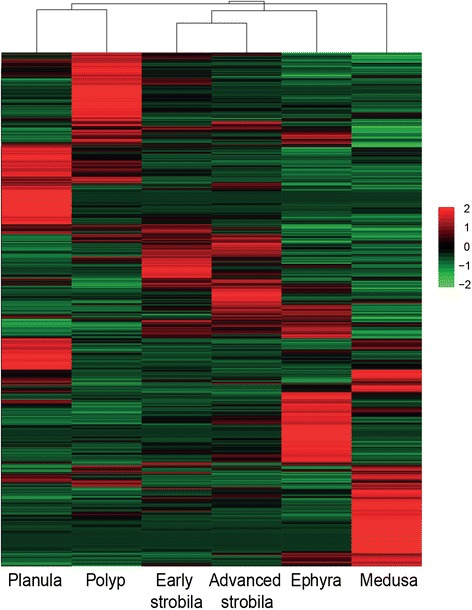
**Heatmap comparing expression of the reduced set of transcripts in the six developmental stages.** The reduced set of transcripts contains the selected 46,657 Trinity transcripts with minimum FPKM of 1.5 in at least one life cycle stage. Intensity of colour indicates expression levels. Similarity between individuals with hierarchical clustering is shown above the heatmap.

Reads from the different life-cycle stages were re-aligned to the assembled transcriptome and in order to visualize the expression patterns of transcripts at the different stages we performed hierarchical clustering (Figure 2). This illustrates the clustering of developmental stages, where that early and advanced strobila clustered together with ephyra in terms of gene expression levels. It also demonstrates the presence of a large number of transcripts with stage-specific expression.

The transcriptome of three stages (polyp, strobila and ephyra) of an *Aurelia aurita* Roscoff strain from a colder geographic region was recently published, using the Roche 454 GS-FLX+ system [13]. Comparison of the transcriptome of our newly assembled *Aurelia* RS strain with that of the published Roscoff strain using dc-megablast (e-value cutoff of 10^−5^ and above 60% identity of sequences larger than 100 bp), showed that about 50% of the transcripts from the Roscoff transcriptome were identified in our RS transcriptome, while 24% of our *Aurelia* RS transcripts were present in the Roscoff dataset (Additional file 4). Thus, as expected, many of the transcripts corresponded between the two species, but our RS strain dataset also included transcriptional data from additional stages and the Illumina dataset provided higher sequence coverage. Both of these will therefore be valuable datasets for the community.

### Functional annotation

We next used Blast2GO to annotate the selected 46,657 Trinity transcripts. We carried out a Blastx search against the NCBI (GenBank) non-redundant protein, and for each result we saved the top 10 hits with e-values of 10^−3^ and lower. Distribution of the blast data can be seen in Additional file 5. Because the ability to find significant sequence similarity depends on the length of the query sequence, shorter assembled Trinity transcripts are less likely to match known genes; indeed, of the 27,632 transcripts without Blastx results about 49% consisted of 200 to 500 bp. However, the larger sequences, without Blastx may be transcripts encoding novel proteins with no similar sequences in the database. The assembled transcripts were assigned sequence names based on the best blast hit for that sequence. We assigned 10,285 unique names, and this was an underestimation of the total number of genes in the reduced dataset given that many sequences lacked blast hits (Additional file 6).

### Patterns in transcript expression across life-cycle stages

**Figure 3:**
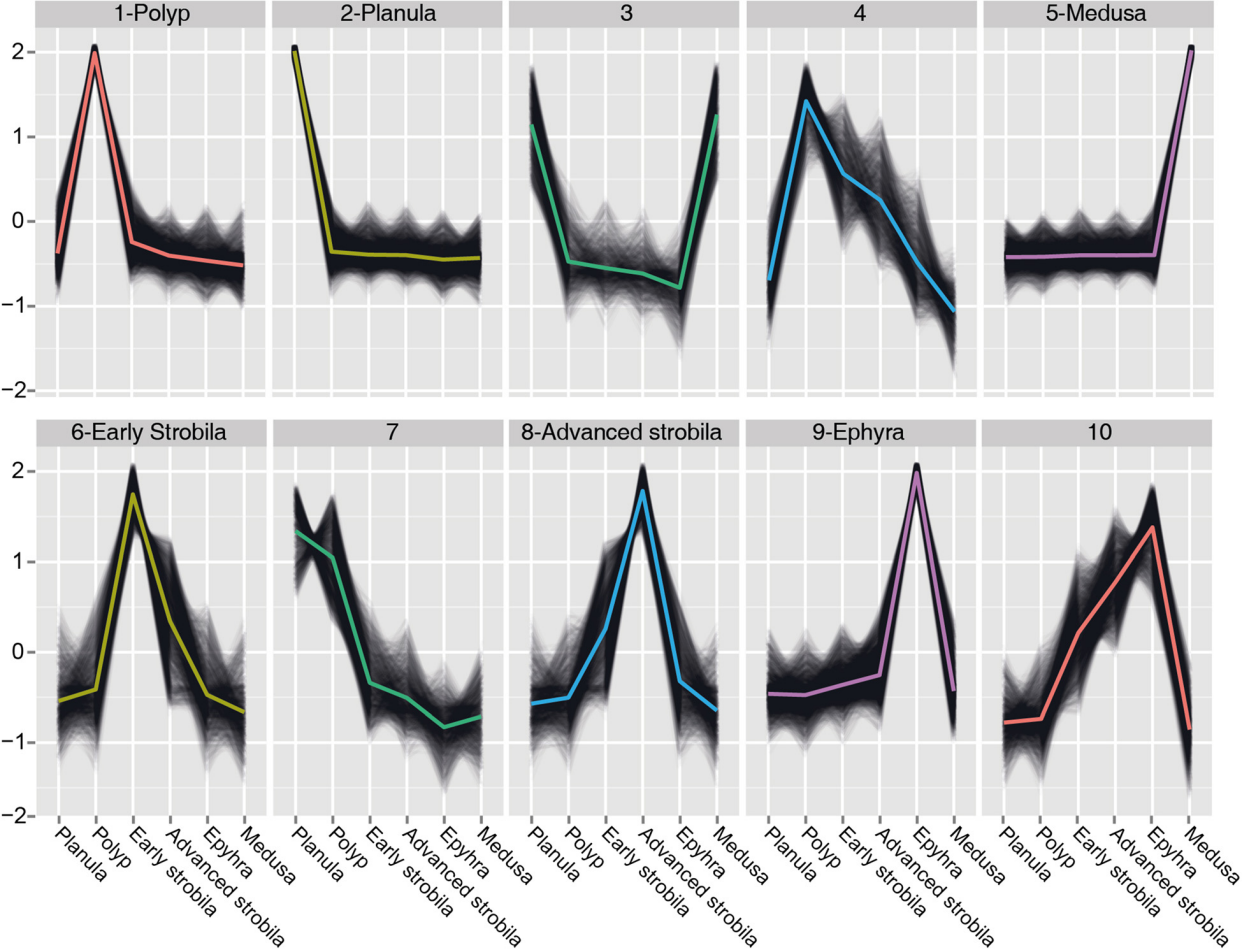
**Clustering profiles.** Plots of k-mean profiles of the reduced set of transcripts with their centroids highlighted in color. The six clusters that were further analysed are marked near the profile number. The vertical axis represents relative transcript abundance, and the horizontal axis depicts the life-cycle stages.

Our next objective was to identify processes for which expression changes during progression of the life cycle. To this end we set out to identify sets of transcripts for which expression patterns across the different developmental stages are coherent. Using k-means clustering of the reduced set of transcripts we identified six clusters each demonstrating stage-specific expression in one of the six life-cycle stages (Figure 3 and Additional file 6), and in these (clusters 1, 2, 5, 6, 8 and 9) we further analyzed only those transcripts with a distance of less than the median distance to the centroid.

**Figure 4:**
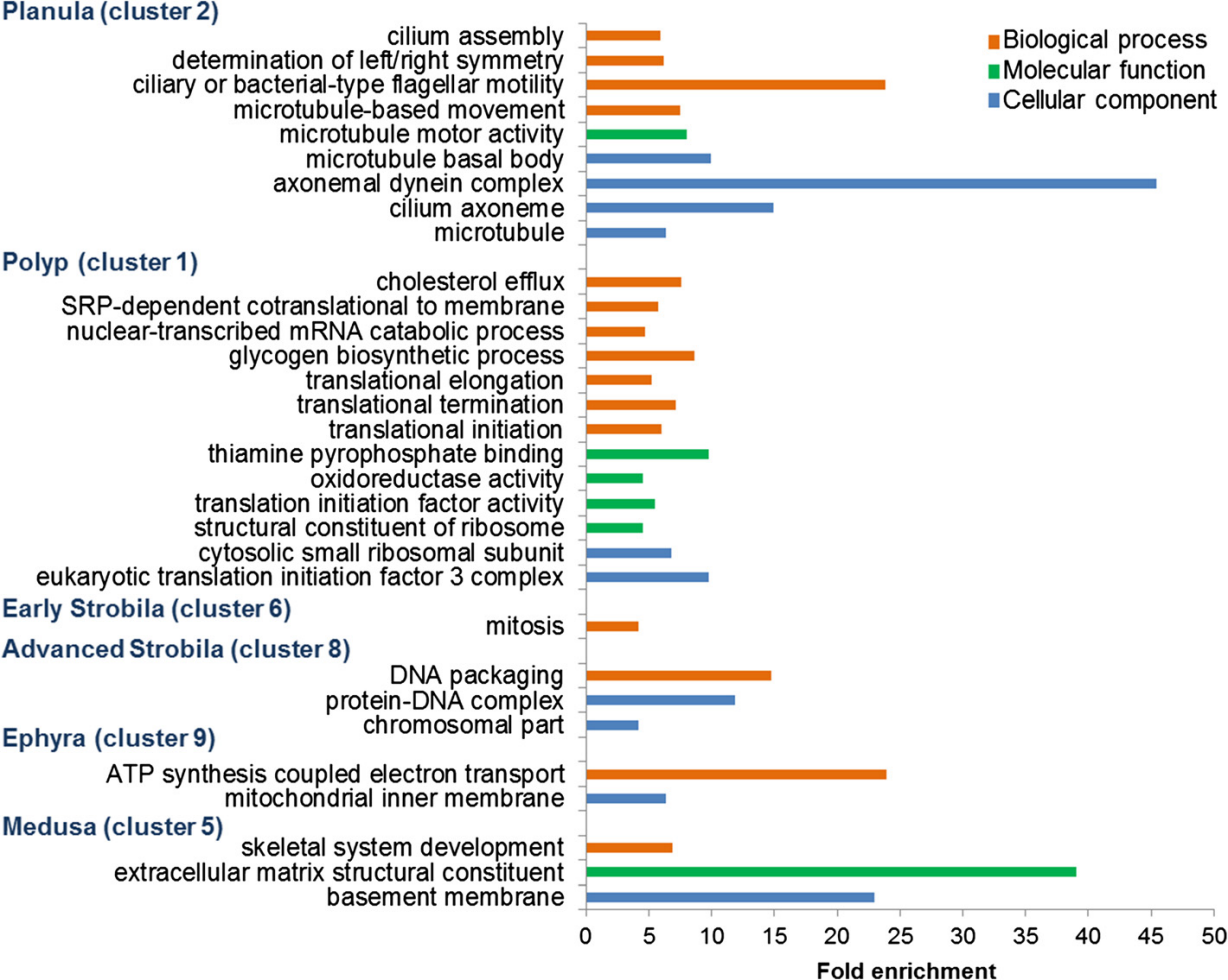
**GO enriched processes in** ***Aurelia*** **life-cycle stages.** Shown are the significantly overrepresented (FDR < 0.05) enrichment factors of GO biological processes (orange), molecular function (green), cellular component (blue), terms in planula, polyp, early strobila, advanced strobila, ephyra and medusa clusters. Only groups containing more than five genes and having a fold enrichment factor >3 are presented. For the complete data see Additional file 7.

**Figure 5:**
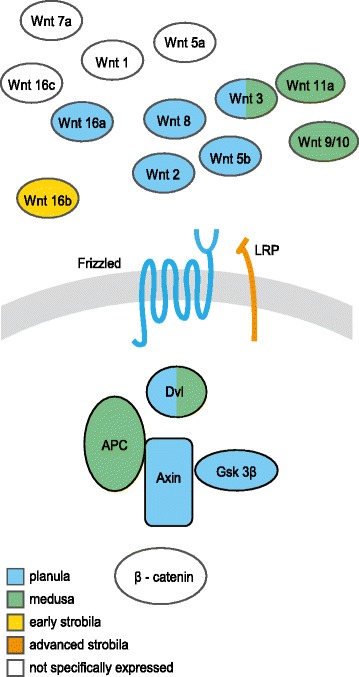
**Model of the expressed Wnt/β‐catenin pathway in the life-cycle stages.** Shown are Wnt related transcripts expressed specifically in clusters of planula, medusa, early strobila and advanced strobila, together with expression of nonspecific Wnt-related transcript cluster expression. APC, adenomatous polyposis coli; Dvl, Dishevelled; GSK3b- glycogen synthase kinase 3β; LRP, LDL-receptor-related protein. For a detailed list of the Wnt related transcripts see Additional file 8.

We first used GO enrichment analysis to search for enriched transcripts with related functions in the six selected clusters (Figure 4 and Additional file 7). In cluster 2, expressed mostly in the planula, the most highly enriched pathway was related to microtubules and cilia. This finding was consistent with the anatomy of the *Aurelia* planula, which has an ectodermal ciliary layer that surrounds its body and provides its means of motility [25]. An additional biological process found to be enriched was related to basic developmental features of left-right symmetry, which were related to kinesin-like 3b protein and other proteins, and are known in mammals to play a role in symmetry determination [26]. Close analysis of these genes in this enriched process enabled us to identify components of the Wnt pathway. Wnt is an important player in cnidarian development and we therefore looked for additional planula transcripts in this pathway. Figure 5 shows that five *Wnts*, *2*, *3*, *5b*, *8* and *16a* [27] as well as *Frizzled*, *GSK-3β* and *Axin* were specifically expressed at the planula stage. Interestingly, *Wnt9/10*, *Wnt11a*, and a transcript variant of *Wnt3* were specifically expressed at the medusa stage, whereas *Wnt 16b* expression was specific to early strobila (Figure 5 and Additional file 8).

The polyp, which represents a sessile stage in the jellyfish life cycle, manifested a housekeeping process of ribosomal activities that included transcripts involved in the translational machinery (Figure 4 and Additional file 7). In addition, energy storage was represented in the form of glycogen synthesis or sugar and amino acid metabolism. In clusters of early and advanced strobilae, in which the polyp tissue changes its shape and size, the most enriched process was mitosis. In ephyra, the released mobile stage, the main processes in the cluster were in the mitochondria and were involved in energy release. Finally, the mature medusa stage, characterized by its prominent bell, contained in its cluster enriched ECM GO terms that included transcripts such as collagen, fibrillin and laminin.

### Most abundant specific transcripts in the six life-cycle stages

The identities of the most abundant transcripts may provide an additional perspective on important processes during the different life-cycle stages. We therefore looked for the 30 most highly expressed transcripts in each developmental cluster (Table 3). In all life-cycle stages except for the polyp, many of the abundant transcripts encoded for unknown proteins.

**Table 3:** **Most abundant transcripts in the different life-cycle stages**

**Planula**	**No.**	**Polyp**	**No.**	**Early Strobila**	**No.**
CDC 20	1	Elongation factor	1	Mam-Tsp1-Cub domain	7
Astacin	2	Actin	1	hnRNP	2
Hydrolase	1	Ribosomal protein	21	Peroxiredoxin	1
REJ domain	1	Translation control	1	AIR carboxylase	1
Cysteine-rich secretory protein	1	Tubulin	1	ShK domain-like	1
CEP63	1	RNA-binding protein	1	Proteinase inhibitor	1
Protease	1	Ubiquitin	2	Unknown	17
Meprin-like astacin	1	Translation initiation	1		
Coagulation factor 5/8 domain	1	Unknown	1		
Unknown	20				
**Advanced Strobila**		**Ephyra**		**Medusa**	
CL390-like	2	Myosin light chain	1	BHMT	1
Ef hand domain	1	Cytochrome C oxidase	1	Collagen alpha1	1
CL112	1	Rdh8	1	Antistasin	2
Histone H2B	1	ATPase subunit	1	Follistatin-like	1
Mam-Tsp1-Cub domain	1	Unknown	26	Lecithin retinol acyltransferase	1
Unknown	24			Myosin light chain	2
				Myosin heavy chain	2
				vWFA Hemicentin-like	1
				ShK domain	1
				ATP synthase gamma	1
				Unknown	17

No.- indicates the number of closely related transcripts that were among the most highly expressed.

In the planula cluster, the most abundant transcripts were those involved in the cell cycle (CDC20 protein, centrosomal protein 63 (CEP63)), cell adhesion (coagulation factor 5/8 domain), peptidase activity, hydrolysis and protein with receptor for egg jelly (REJ) domain. Most of the highly enriched transcripts expressed in the polyp cluster were ribosomal proteins and transcripts related to translation and the cytoskeleton.

In the early strobila cluster most of the highly expressed transcripts were transcript variants of unknown proteins containing Mam-Tsp1-Cub domains. These are usually extracellular domains that may function in cell adhesion, cell signalling and development. Additional highly expressed transcripts were related to purine metabolism (AIR carboxylase), peroxisome antioxidant and proteinases. The strobila cluster was recently characterized using a Roscoff *Aurelia* strain [13] and was shown to express several unique *Aurelia* transcripts including CL390 and CL112, named by Fuchs et al. [13]. In the advanced strobila cluster we identified two highly expressed transcripts with marked similarity to CL390 (68% protein identity) and CL112 (89% protein identity). However, although the CL390-like protein of the RS strain was composed of many arginine repeats and contained the putative secreted signal peptide similar to that found in the Roscoff strain, it did not contain the short 7-amino-acid sequence with two tryptophan residues previously shown to be active in strobilation induction. Instead, an additional tryptophan residue was found at the C terminus of the peptide (Additional file 9). On the other hand, 5-methoxy-2-methyl-indole, suggested to mimic the effect of the CL390 short peptide [13], induced strobilation in our RS strain with similar kinetics to those of the Roscoff strain (data not shown).

In the ephyra cluster we identified transcripts related to the respiratory chain and ATP synthase, in line with the GO categories of mitochondria and ATP electron transport found to be enriched at this stage. Additional highly expressed transcripts were related to motor proteins such as myosin, and to retinoic acid metabolism such as all-trans-retinol dehydrogenase 8 (rdh8). Collagen alpha 1, as the main component of the medusa bell, was one of the most abundant transcripts at the medusa stage, together with the von Willebrand factor type A domain (vWFA) that resembles hemicentin, an ECM protein. Also detected were additional muscle function-related motor proteins such as myosins and follistatin-like proteins as well as betaine-homocysteine methyltransferase (BHMT), a metallotransferase in the amino acid synthesis pathway and antistasin, a serine protease inhibitor.

### Stage-specific transcription factors

**Figure 6:**
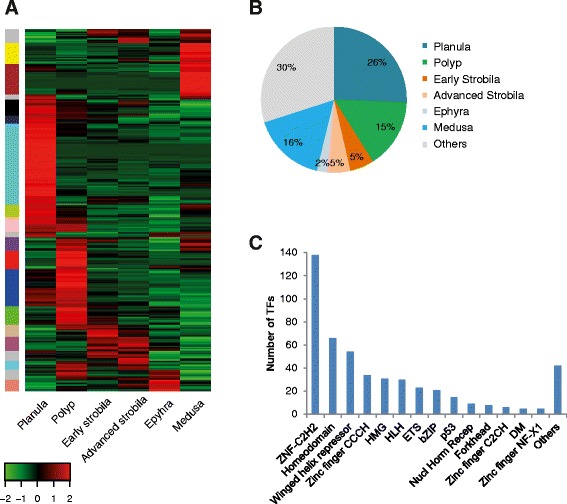
**Transcription factor expression in the different** ***Aurelia*** **life-cycle stages. (A)** Hierarchical clustering of TFs found in the reduced transcriptome set. Color code on the left represents their clade group, indicating clusters of TFs with similar expression levels (see Methods). The full list of TFs (and their accompanying color code) can be found in Additional file 10. **(B)** Percent of TF expression at each stage cluster. **(C)** Classification of TFs according to their family group [75]. Families with fewer than five members were classified as ‘others’.

Transcription factors (TFs) are important regulators of gene expression and controlling cellular and developmental processes. We therefore looked for the TFs expressed at the six life-cycle stages (Figure 6). We identified 487 genes encoding putative TFs that could be divided into 31 gene families (Figure 6 and Additional file 10). Of these TFs, the largest group of 126 putative transcription factors (26%) was specifically expressed in the planula cluster (Figure 6). These included TFs known to play important roles during early development in other organisms [28] such as *sox c* from the high mobility group box (HMG-box), a hypoxia-inducible factor 1 alpha (*hif1α*), and other TFs from the helix-loop-helix (HLH) family, t-box 2 from the p53 family, *coe1* from the IPT/TIG family and others. Also specifically expressed in the planula cluster was the forkhead box protein J1 (*foxj1*) which is known to be involved in ciliogenesis across diverse groups of metazoans [29,30].

The smallest group of stage-specific TFs was found in the ephyra cluster, and consisted of 11 transcripts (2%) including the atonal homolog-like (*Atohl*), which controls photoreceptor development in Bilateria [31]. This small number of TFs may be due to the ephyra transcripts expression levels clustering together with the early and advanced strobila transcripts (see Figure 2) and therefore TFs for which expression is shared by these three stages will not be cluster-specific. In the cluster of the early strobila stage we identified the retinoid X receptor (*RxR*) which was also expressed, though to a lower extent, in the strobila advanced cluster, in addition to kruppel-like factor 13 (*klf13*), and others. TFs specifically expressed in the advanced strobila cluster were *c-jun-like*, *fox*, *dmrt1* and others. In the medusa cluster, 78 TFs (16%) were expressed in a stage-specific manner, including *sox* genes, hepatocyte nuclear factor 4, another nuclear receptor and more (Figure 6 and Additional file 10). In the polyp cluster the most highly expressed TFs were related to the translational machinery: four subunits showed high expression of the eukaryotic initiation factor 3 together with CCAAT-enhancer binding protein β.

Of particular interest was the homeodomain group known to be involved in developmental regulation (Additional file 11). We found 18 TFs from the homoeodomain group in the planula cluster, including *Hox 9–14* and *Otx1-like*, whereas in the polyp cluster members of the *lim* class and those related to aristaless were expressed (Additional file 11). During the early strobila stage there was notable expression of barh-like 2, known to play a role in the eye and external sensory organs of *Drosophila* and retina and sensory neurons in vertebrates [32], though it was also expressed in the polyp. The advanced strobila cluster showed specific expression of the dorsal root ganglia (*drg*) homeobox gene. In the ephyra cluster msh homeobox-like 2 was expressed, a TF that plays a role in muscle segmentation in *Drosophila*. In the medusa cluster additional *Hox* genes as *Hox 1*, *GSX* and *NK2b* were expressed. Overall, a significant proportion of TFs showed stage-specific expression.

## Discussion

In the present study, comparative expression profiling using the Illumina sequencing platform and de-novo transcriptome assembly using Trinity was performed on six life-cycle stages of *Aurelia*. This is the first transcriptomics analysis which spans the full life cycle of a scyphozoan jellyfish and our findings demonstrated that each stage in the life cycle has a unique expression profile, possibly guided by the activities of stage-specific TFs.

### The planula stage

The motile planula which emerges from the fertilized egg on completion of embryogenesis, serves as the main body for axis determination and morphogenesis, and must find an appropriate ecological niche in which to settle and transform into a sessile polyp. Planula motility is achieved by an ectodermal ciliary layer that surrounds its body [25]. The finding of cilia-related processes in our GO enrichment analysis of planula-specific transcripts is in line with the expression patterns associated with motility recently characterized in the planula of the coral *Acropora digitifera* [33]. Moreover, *foxj1*, an F-box TF and a key regulator of motile ciliated cell differentiation programs in different model organisms such as flatworm, fish and mammalian systems [29,30,34], was found here to be specifically expressed at the planula stage.

Cell division and differentiation likely continue during planula development, as evidenced by high expression of CDC20 and CEP63, conserved essential regulators of the cell cycle in metazoans [35,36]. Moreover, among the most abundantly expressed genes in the planula were several proteases and specifically astacins, members of a large family of zinc metalloproteases. Astacins have diverse physiological functions ranging from digestion and hatching to morphogenesis and pattern formation [37]. The two astacins identified here belong to two subgroups, one containing secreted astacins and the other is a membrane-bound, meprin-like astacin [38]. Secreted astacins have been suggested to participate in proteolytic processing during early invertebrate and vertebrate development, and were shown to be involved in digestion in the mature hydromedusa *Podocoryne carnea* [38-40]. In addition, in cnidarians of class Hydrozoa, astacins were detected in sites related to morphogenesis [38-40], whereas meprins were shown to play a role in tissue differentiation and signaling and a meprin-like astacin was suggested to function in morphogenesis of the hydra foot [37,41]. We suggest that astacins in the *Aurelia* planula play both roles: one has to do with the proteolytic processing of storage proteins with the aim of supporting planula development before metamorphosis, as the gastric cavity opening and mouth have not yet developed, and the other function in the process of morphogenesis, which prepares the planula for its transition to the polyp shape.

The relatively large percentages of planula-specific TFs signify both the complexity and the importance of this stage in establishing the organism’s body. This was highlighted by the many members of the homeodomain TF family, including Hox members that play a critical role in developmental patterning and were found here to be specifically expressed at the planula stage. The homology and function of bilaterian *Hox* genes in the process of cnidarian morphogenesis along the anterior-posterior (AP) axis is still debatable [42-45], with a recent study demonstrating of an ancestral regulatory module that may have been recruited for AP patterning in Bilateria [46]. We found that in the Hox group, only *Hox9-14* genes were expressed in the planula and the others *Hox1* and *GXT* were expressed at the medusa stage, as described in the hydromedusa *Clytia* [42].

In addition to the TFs related to planula development we also identified *hif1α*, an environmentally-induced TF that is highly expressed at the planula stage. *hif1α* is a conserved TF in all Metazoa and mediates gene expression involved in a range of metabolic and developmental processes under hypoxic conditions [47]. Oxygen availability is critical in marine habitats, as its content in the water can change rapidly as a result of oxygen consumption by organisms and of environmental pressures such as temperature rise and organic pollution. Hypoxia was suggested to promote outbreaks of jellyfish blooms since these organisms are less affected than their prey by oxygen deficiency [3,12]. In addition, hypoxic conditions were found to accelerate *Aurelia* planula settlement and polyp survival in natural conditions [12], and *Aurelia* polyps and medusae were recently shown to react to hypoxia by increasing their expression of *hif1α* [48]. Our finding of specific expression of *hif1α* in the planula, possibly indicative of oxygen stress conferred on jellyfish in captivity, is in line with previous findings and may suggest that *hif1α* plays a central role in maintaining and regulating the normal cellular functions needed for successful planula transformation.

The Wnt signaling pathway has a conserved role in axial patterning throughout Metazoa, and has been suggested to play a role in oral-aboral patterning in Cnidaria [14,49-51]. We have previously identified the Wnt ligands members in *Aurelia* and demonstrate that they are grouped with the medusozoan cluster [27]. Here we show that the Wnt pathway is expressed mainly in the planula stage and that several Wnt ligands are specifically expressed at the early strobila and the medusa stages. Scyphozoan Wnt ligands have not yet been thoroughly studied and it will be interesting to compare their spatial expression to that of other cnidarians and specifically to hydromedusae in order to better understand their function.

### The polyp stage

The polyp is the perennial sessile phase in the scyphozoan life cycle. After planula metamorphosis, the polyp actively feeds and this serves as the main stage in which asexual proliferation takes place, either by budding to form more polyps or by strobilation to form the ephyrae that will eventually develop into medusae. As this is a long lasting stage, most of the enriched processes were found to be related to common basic cell activities such as translation machinery and glycogen metabolism.

### Early and advanced strobila stages

In the strobilation process the polyp undergoes substantial morphogenetic changes. At early strobilation, initial transverse constrictions occur that further develop in the advanced strobila into well-separated tissue that eventually give rise to a completely independent mobile organism, the ephyra. During early strobilation, the most abundant transcripts encoded an *Aurelia*-specific protein containing a cub domain that is usually associated with developmentally regulated proteins [52]. This cub-domain protein was also abundant in advanced strobila, as were two additional *Aurelia*-specific peptides, CL112 and CL390-like, recently characterized in the *Aurelia* Roscoff strain [13]. CL390 was shown to induce strobilation in the *Aurelia* Roscoff strain and was suggested to become biologically active after being processed into small peptide fragments. Surprisingly, our *Aurelia* RS strain did not contain the same small suggested peptide fragment sequence, but it is possible that other changes occurring in the *Aurelia* RS CL390-like sequence provide the conformation needed for its biological activity. In line with these findings, we found that 5-methoxy-2-methyl-indole, the pharmaceutical compound that was shown to mimic this conformation [13], induced strobilation in our *Aurelia* polyp cultures. In both the early and the advanced strobila we detected up-regulation of *RxR* that was previously shown to play a role in strobilation induction and was related to CL390 regulation [13]. Additionally, the conserved binding function of *RxR* to regulatory elements was shown in the cubozoan jellyfish *Tripedalia cystophora* [53].

Our search for additional early-strobilation specific TFs revealed the potential involvement of *klf13*, a member of the KLF TFs that participate in diverse cellular development processes such as cell differentiation, proliferation, cell growth, and apoptosis [54]. As strobilation advanced, additional TFs such as the paired-like homeobox gene *drg* were expressed. Expression of *drg* is restricted to the peripheral nerve system and plays a role specifically in somatosensory functions, indicative of mechanoreceptive neuronal function in invertebrates and vertebrates [55,56]. In our study its expression was up-regulated in the advanced strobila and then down-regulated in the ephyra. This transient expression suggests the *drg* might be involved in specifying the future sensory system in the next stage of the life cycle, the ephyra [57].

Our data suggest that relatively few TFs operate specifically during the strobila phase. This is surprising as such a dramatic morphogenetic process could be expected to involve many more TFs. However, unlike the transformation from planula to polyp, the strobilation process is carried out from a fully developed stage. Thus, it is possible that the necessary TFs are expressed locally and cannot be traced using the whole-organism transcriptome analysis.

### The ephyra stage

Once the ephyra is released from the strobila it has to maintain its balance and motility in the water column and undergo additional differentiation and growth to achieve the final medusa shape. The continuous pulsing and movement of the ephyra require energy, and our finding that the main biological process and the most abundant proteins were related to the respiratory chain and energy was consistent with this demand. In concert with development of the free-swimming ephyra the rhopalia, containing photoreceptors and mechanoreceptors, begin to develop [58]. Interestingly, among the abundant transcripts we identified a putative *rdh8* homolog, which in bilaterians plays an important role in the visual cycle and is located in the photoreceptor outer segment [59]. In addition, we showed that *Atohl* TF of the HLH family was specifically expressed at the ephyra stage. The Atonal genes were first identified in *Drosophila* and are strongly linked with the specification of photo- and mechanoreceptor cells [31]. In vertebrates, *Atoh7* is required for retinal ganglion cells differentiation and *Atoh1* for mechanosensitive cells development [60]. In the hydrozoan jellyfish *Podocoryne carnea*, *Atohl1* was found to be expressed in mechanosensory cells and neuronal precursors [61]. Our finding that *Atohl* gene is also highly expressed specifically in the ephyra when the sensory machinery is developing may point to conservation of its role in the Scyphozoa. It is likely that *Atohl1* may be also expressed in the sensory cells at the adult stage, but these cells were not sampled in the study.

### The medusa stage

The medusa stage was characterized from the central bell tissue and therefore the results represent genes of the specific tissues sampled from the medusa and not genes expression from the whole medusa. The medusa bell is composed mainly of mesoglea an acellular layer of ECM. This thick layer maintains the jellyfish body structure, morphogenetic processes and enables its locomotion [62]. Many studies have shown that cnidarian ECM harbors proteins similar to bilaterian ECM [63-66]. We found that fibrillar collagen 1 and a vWFA domain with similarity to hemicentin protein are the most highly expressed ECM transcripts at the medusa stage. Fibrillar collagens comprise most of the ECM matrix in Bilateria [63] and hemicentins were shown to have many functions including maintenance of tissue integrity by adhesion or by providing an elastic fiber-like structure [67]. The vWFA domain, however, is found in many extracellular proteins and more research is needed to identify its function in the medusa ECM. The unique mode of locomotion in the medusa requires not only an elastic ECM but also motor power, which is derived from striated muscles that propel the medusa by rapid contractions. The most abundant transcripts were indeed found to contain myosin heavy and light chains, together with folistatin-like protein, suggested to participate in the regulation of muscle growth in mice [68].

Interestingly, we found that that one of the most abundantly expressed transcripts was *BHMT (betaine-homocysteine methyltransferase)*, which catalyzes the resynthesis of methionine using homocysteine and the methyl donor betaine. BMHT is the only enzyme known to use betaine and because of the important role of betaine in maintaining cellular volume by increasing water retention of the cells and replacing inorganic salts, BHMT activity helps to maintain cellular osmolytic equilibrium [69,70]. It is tempting to speculate that BHMT plays a similar role in regulation of osmotic changes in the medusa bell to its roles in mammalian liver and kidney. BHMT was also found to be a major protein in the soluble crystalline fraction of the rhesus monkey lens [71], suggesting additional unknown roles for this protein. Future analyses of BHMT cellular expression pattern may provide more clues to its function.

## Conclusions

*Aurelia* are distributed worldwide and have an enormous effect on marine ecosystems. Their complex life cycle incorporates sexual reproduction, asexual proliferation and sequential metamorphic events resulting in sessile polyps and motile ephyrae. In this study we used an unbiased approach of RNA-Seq with de novo Trinity assembly to characterize the expression profiles of six life-cycle stages spanning the full developmental trajectory of *Aurelia.* We found stage-specific molecular pathways and identified specific TFs underlying regulation of the different life-cycle stages. The large variety of biological and molecular processes found to be specifically expressed in the various life-cycle stages revealed the transcriptomic complexity and adaptation during the *Aurelia* life cycle. Our study provides the first full life-cycle transcriptomic catalogue of a scyphozoan and reveals potentially key genes and pathways that participate in the morphological, physiological and functional changes that take place during the *Aurelia* life-cycle.

## Methods

### Culturing conditions

*Aurelia aurita* planulae were isolated from mature jellyfish, collected from the Red Sea in Eilat, Israel. In order to get polyps, the planulae were kept in natural sea water for about 2 weeks until transformation to polyps took place. Following transformation, polyps were cultured in 33 ppm artificial sea water (Red Sea salts) and at 18°C they reproduced asexually without strobilation. Induction of strobilation was achieved by raising the polyp incubating temperature to 25°C. Polyps were fed twice weekly with freshly hatched *Artemia nauplii*.

### Sample preparation and sequencing

mRNA from the five developmental stages, other than the medusa, was isolated from whole organisms (hundreds of individuals). mRNA from the medusa stage was isolated from several specimens during spring 2012, and only the middle part of the bell was extracted using Tri-Reagent Kit (Sigma). Samples were prepared for sequencing using Illumina’s TruSeq RNA Library Prep Kit (Illumina) according to the manufacturer’s instructions. Six samples were sequenced using 100-bp paired-end reads on an Illumina HiSeq2000 lane and TruSeq v3 flow chamber at the Life Sciences and Engineering Infrastructure Unit, Technion, Haifa.

### Transcriptome assembly

The RNA-Seq data was de novo assembled using Trinity (version trinityrnaseq_r2012-04-27) [20,21] combining pooled Illumina reads from all developmental time points. Contigs shorter than 200 bp were eliminated. Contamination was identified by blasting (Blastn) the contigs against sequences of possible contaminating species such as bacteria or *Artemia*, the latter serves as *Aurelia*’s food source. Contigs with successful hits (E-value cut-off of 1e^−6^ and >95% identity) were removed from the assembly before further analysis. To assess the completeness of the transcriptome assembly, the CEGMA (Core Eukaryotic Genes Mapping Approach) software was applied [23]. To examine the contiguity [24] of the Trinity assembly, the number of full-length transcripts was assessed according to transcript sequence representation of homology to known proteins, as previously described [21]. The Aurelia transcripts were searched against the *Hydra magnipapillata* protein NCBI database (ftp://ftp.ncbi.nlm.nih.gov/genomes/Hydra_magnipapillata/Gnomon/) using BLASTX with an E-value cut-off of 1e^--20^. Those transcripts matching at least 70% of the length of the top-matching protein sequence were identified and tabulated. The assembly has been submitted to NCBI’s Transcriptome Shotgun Assembly (TSA) Sequence Database under the accession number GBRG01000000.

### RNAseq analysis and clustering

RSEM (RNA-Seq by Expectation-Maximization) was used to estimate transcript abundance from each sample separately using the standard settings, and the obtained expression values were TMM (Trimmed Mean of M-values) normalized [72]. Read counts were then converted to Count per Million (CPM) values using EdgeR [73]. For heatmap generation, CPM values were scaled as Z-scores for each tested transcript. Heatmaps were clustered using the flashClust R packge with complete linkage clustering based on Pearson correlation. Transcript clade groups were detected using the cutreeStaticColor function of WGCNA [74]. The above scaled data were also used for k-means clustering analysis using k = 10. For each k-means group, the Euclidean distance from the centroid was calculated for each gene, and only those genes for which the distance from the centroid was smaller than the median distance were analysed further.

Using dc-megablast, we subjected the full RS transcriptome to similarity search against the Roscoff transcriptome [13] allowing one best hit at an e-value cutoff < 1e^−7^ and > 60% identity of sequences larger than 100 bp.

### Transcriptome annotation and analysis

Transcripts with TMM-normalized FPKMs of minimum ≥ 1.5 (46,657 transcripts) were annotated with Blast2GO (http://www.blast2go.com/) (July 2012), using Blastx against the non-redundant protein database with an e-value cutoff < 1e^−3^. In addition, we applied Trinotate (http://trinotate.github.io/) to annotate the transcripts. The expressed transcripts in the six k-means clusters showing stage-specific expression were tested for enrichment against GO terms (biological processes, molecular function, and cellular components) using Blast2GO with the reduced transcriptome as background and the Fisher exact test (FDR < 0.05). TFs were identified by Interpro according to their family groups as described before [75].

### Availability of supporting data

Raw sequence reads can be found in the SRA database under BioProject PRJNA252562. The Transcriptome Shotgun Assembly project has been deposited at DDBJ/EMBL/GenBank under the accession GBRG00000000. The version described in this paper is the first version, GBRG01000000. The CL390-like sequence can be found in the GenBank under the accession number KM587721.

## Additional files

Additional file 1:
**Phylogeny of** ***Aurelia*** **species based on 16S sequences.** A Maximum Likelihood (ML) tree is shown, with numbers representing bootstrap support in ML and Neighbour Joining (NJ) trees. Eleven sequences were analysed including seven sequences representing *Aurelia* clades described by Schroth et al. [19]: Arabian peninsula clade representing the Red sea and the Persian Gulf (AF461402), *A. limbata* (AF461403), *A. labiata* ( AF461401), Tethys sea clade representing the Mediterranean sea (AF461400), tropical (AF461404), ubiquitous (AF461398), Boreal (AF461399) and White sea strain (KC767899), Baltic sea strain (KC767897), Roscoff strain (KC767898) and Eilat strain (this study, KP144282). Phylogenetic analysis was performed using the MEGA5 software [76]. For the ML tree, the Tamura-Nei model [77] was used, with discrete Gamma distribution to model evolutionary rate differences among sites (5 categories (+G, parameter = 0.3806)). The NJ tree was created using the Maximum Composite Likelihood method [78]. Additional file 2:
**CEGMA completeness report for the transcriptome assembly.**
Additional file 3:
**Contiguity analysis of the transcriptome assembly.** The histogram represents the percent of hydra proteins covered by Aurelia transcripts at different levels of contiguity. Additional file 4:
**Comparison of transcriptomes in** ***Aurelia aurita*** **strains.** Comparison of the *Aurelia* RS strain (red) and the published *Aurelia* Roscoff (R) strain [13] transcriptomes. **(A)** Full transcriptome **(B)** Reduced transcriptome set. Sequence homology (%) in the two strains is indicated according to their color code. Additional file 5:
**Distribution of Blast2GO data.**
Additional file 6:
**Data matrix of reduced transcriptome set.** The expression count data, cluster mapping, and annotated results. Additional file 7:
**GO function analysis table.** The full GO enriched processes in Aurelia life-cycle stages. Additional file 8:
**Wnt/β‐catenin table.** The list of the Wnt related transcripts presented in Figure 5 with their FPKM expression. Additional file 9:
**Comparison of the** ***Aurelia*** **Roscoff CL390 deduced protein sequence with that of the** ***Aurelia*** **RS strain.** The putative signal peptide sequence is underlined, arginine repeats are highlighted in grey, tryptophan residues are highlighted in yellow, and the seven amino acids shown to induce strobilation in the Roscoff strain are marked in red. The sequence of CL390-like was found in a misassembled contig and therefore it was isolated by PCR from strobila RNA and sequenced. The CL390-like nucleotide sequence can be found in the GenBank under the accession number KM587721. Additional file 10:
**Table of TFs expressed in the six life-cycle stages.** The list of the TFs presented in Figure 6 with their family group and FPKM expression. The file order is similar to that shown in Figure 6 and the color code is marked. Additional file 11:
**Heatmap of Homeodomain TF expression in the different** ***Aurelia*** **life-cycle stages.** Hierarchical clustering of homeodomain TFs found in the reduced transcriptome set. The contigs number and the transcript annotations are shown.

## Abbreviations

RS: Red Sea
ECM: Extra cellular matrix
TFs: Transcription factors
GO: Gene Ontology
