# Supplementary figures and images for: Transcriptome profiling of the dynamic life cycle of the scypohozoan jellyfish Aurelia aurita
Source: BMC Genomics. 2015 Feb 14;16(1):74. doi: 10.1186/s12864-015-1320-z (PMC4334923; doi:10.1186/s12864-015-1320-z)

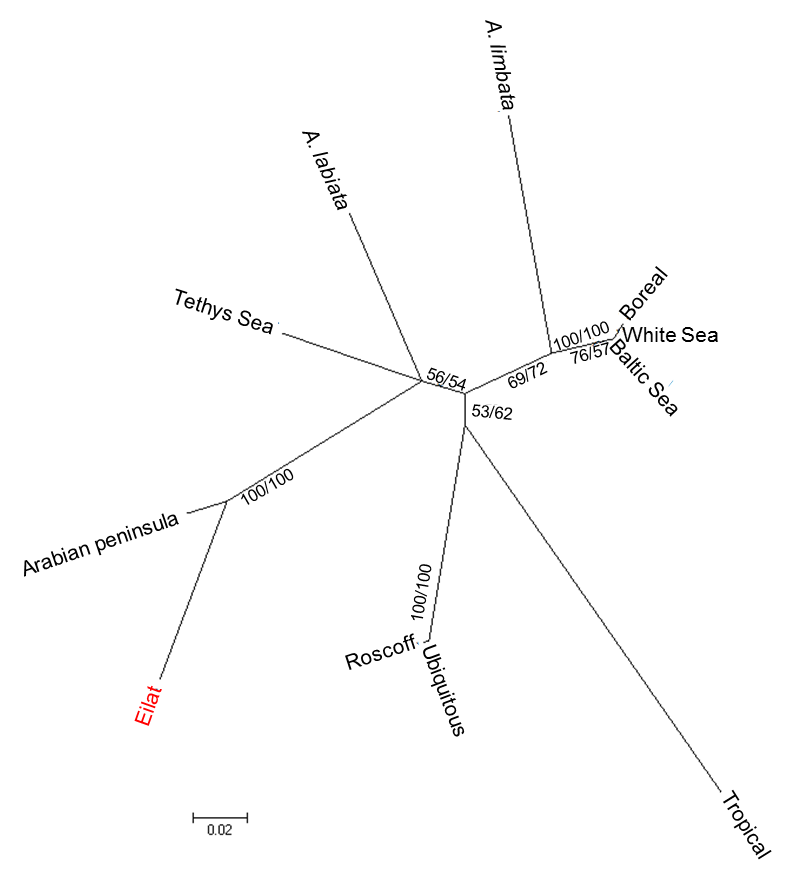

Supplement: Additional file 1: — Phylogeny of Aurelia species based on 16S sequences. A Maximum Likelihood (ML) tree is shown, with numbers representing bootstrap support in ML and Neighbour Joining (NJ) trees. Eleven sequences were analysed including seven sequences representing Aurelia clades described by Schroth et al. [19]: Arabian peninsula clade representing the Red sea and the Persian Gulf (AF461402), A. limbata (AF461403), A. labiata ( AF461401), Tethys sea clade representing the Mediterranean sea (AF461400), tropical (AF461404), ubiquitous (AF461398), Boreal (AF461399) and White sea strain (KC767899), Baltic sea strain (KC767897), Roscoff strain (KC767898) and Eilat strain (this study, KP144282). Phylogenetic analysis was performed using the MEGA5 software [76]. For the ML tree, the Tamura-Nei model [77] was used, with discrete Gamma distribution to model evolutionary rate differences among sites (5 categories (+G, parameter = 0.3806)). The NJ tree was created using the Maximum Composite Likelihood method [78]. [file 12864_2015_1320_MOESM1_ESM.zip › 6073245671439183_add1.bmp]

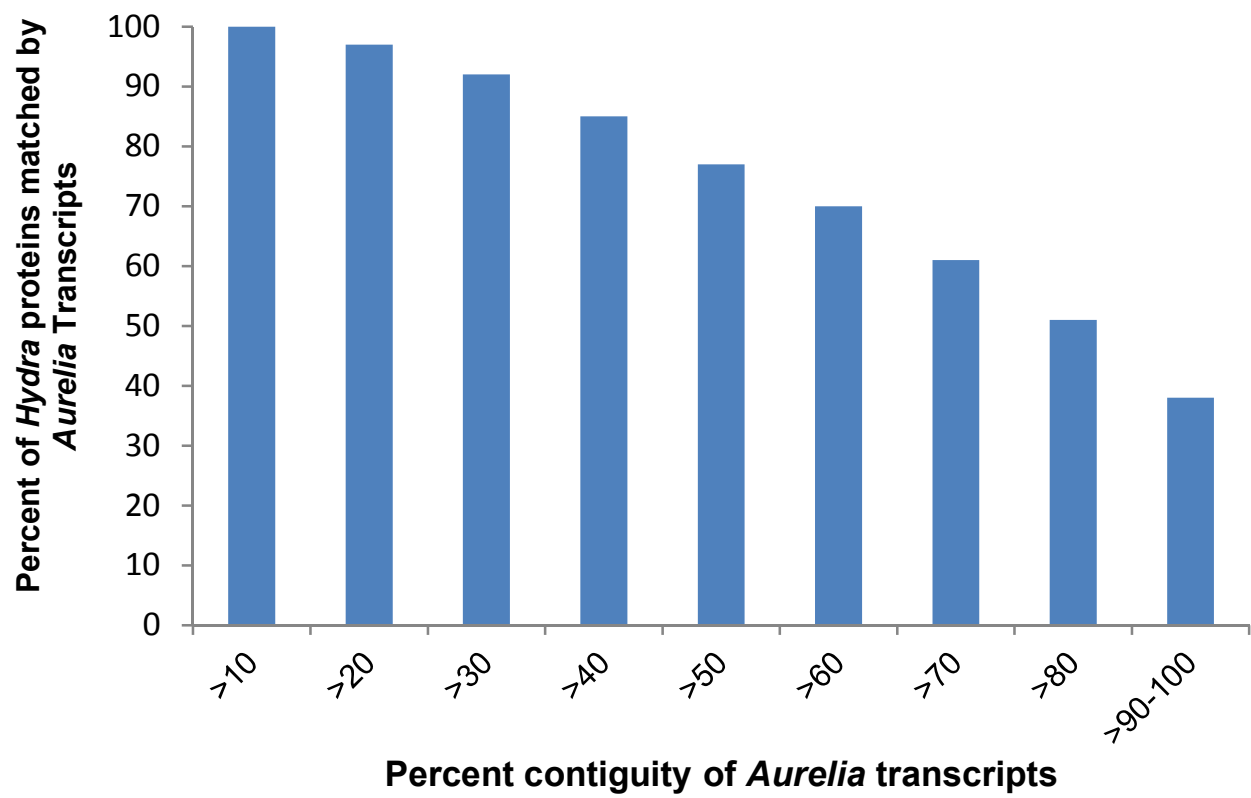

Supplement: Additional file 3: — Contiguity analysis of the transcriptome assembly. The histogram represents the percent of hydra proteins covered by Aurelia transcripts at different levels of contiguity. [file 12864_2015_1320_MOESM3_ESM.pdf]

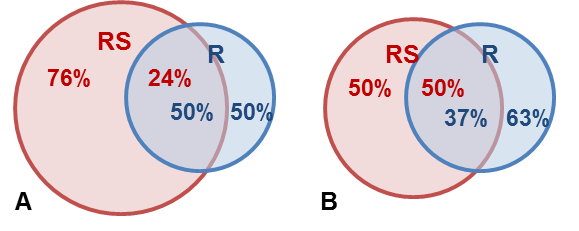

Supplement: Additional file 4: — Comparison of transcriptomes in Aurelia aurita strains. Comparison of the Aurelia RS strain (red) and the published Aurelia Roscoff (R) strain [13] transcriptomes. (A) Full transcriptome (B) Reduced transcriptome set. Sequence homology (%) in the two strains is indicated according to their color code. [file 12864_2015_1320_MOESM4_ESM.zip › 6073245671439183_add4.bmp]

# Data Distribution

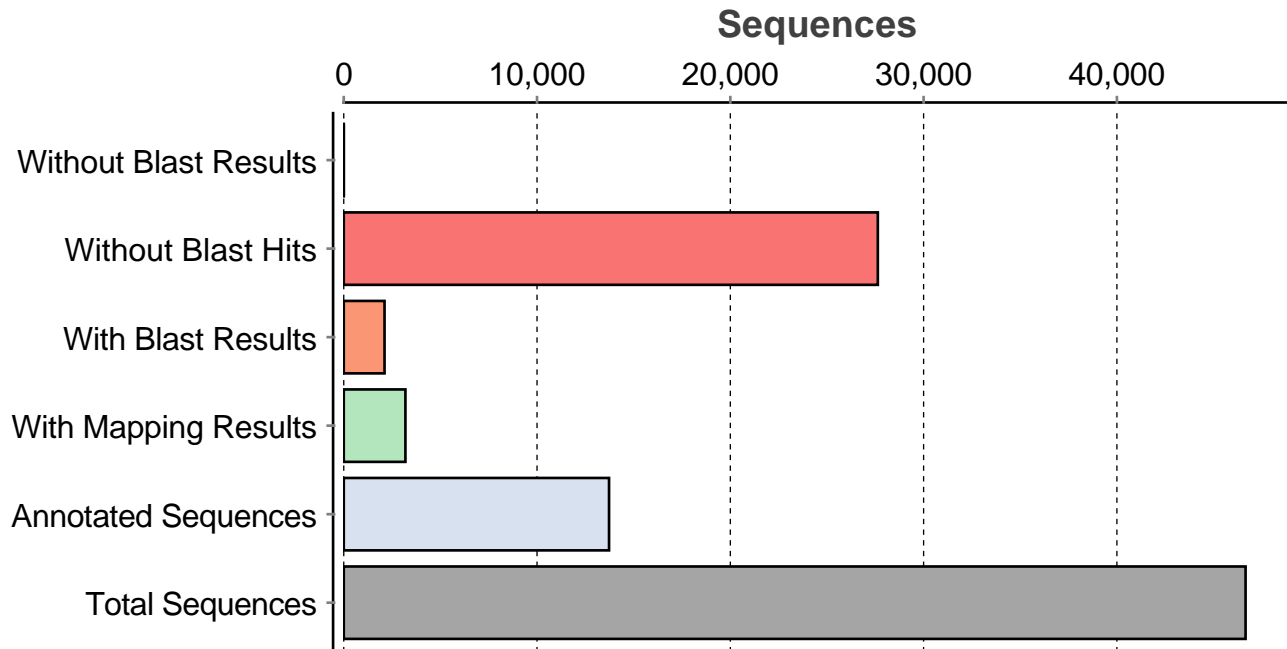

Supplement: Additional file 5: — Distribution of Blast2GO data. [file 12864_2015_1320_MOESM5_ESM.pdf]

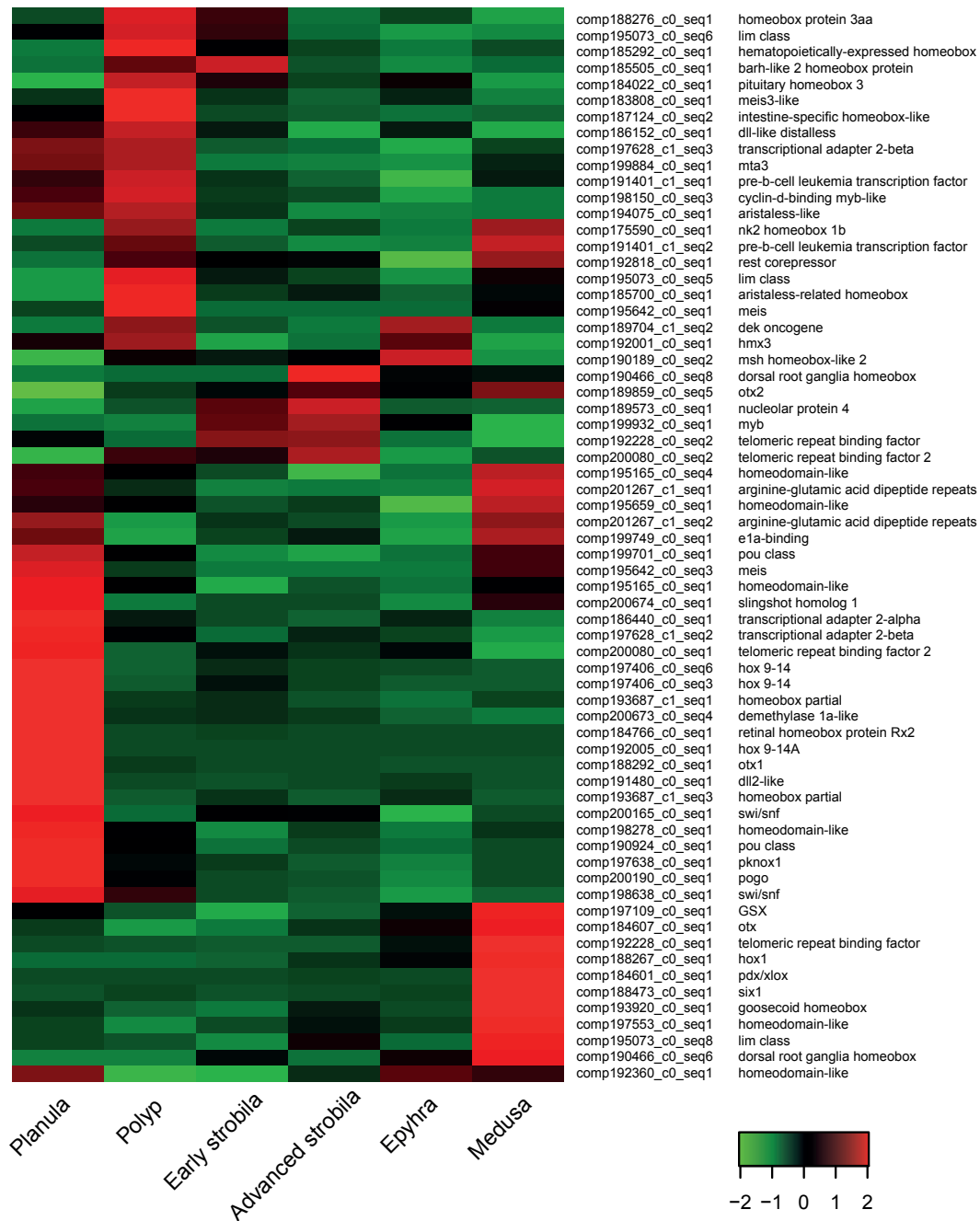

Supplement: Additional file 11: — Heatmap of Homeodomain TF expression in the different Aurelia life-cycle stages. Hierarchical clustering of homeodomain TFs found in the reduced transcriptome set. The contigs number and the transcript annotations are shown. [file 12864_2015_1320_MOESM11_ESM.pdf]
