# Supplementary material for: Transcriptome profiling of the dynamic life cycle of the scypohozoan jellyfish Aurelia aurita
Source: BMC Genomics. 2015 Feb 14;16(1):74. doi: 10.1186/s12864-015-1320-z (PMC4334923; doi:10.1186/s12864-015-1320-z)
Supplement: Additional file 2: — CEGMA completeness report for the transcriptome assembly. [file 12864_2015_1320_MOESM2_ESM.pdf]

Statistics of the completeness of the genome based on 248 CEGs

	#Prots	%Completeness	-	#Total	Average	%Ortho
Complete	228	91.94	-	619	2.71	71.05
Group 1	57	86.36	-	161	2.82	73.68
Group 2	54	96.43	-	151	2.80	79.63
Group 3	55	90.16	-	145	2.64	65.45
Group 4	62	95.38	-	162	2.61	66.13
Partial	235	94.76	-	714	3.04	77.02
Group 1	60	90.91	-	177	2.95	75.00
Group 2	54	96.43	-	178	3.30	88.89
Group 3	58	95.08	-	171	2.95	72.41
Group 4	63	96.92	-	188	2.98	73.02

These results are based on the set of genes selected by Genis Parra

Key:
Prots = number of 248 ultra-conserved CEGs present in genome
%Completeness = percentage of 248 ultra-conserved CEGs present
Total = total number of CEGs present including putative orthologs
Average = average number of orthologs per CEG
%Ortho = percentage of detected CEGS that have more than 1 ortholog

Listing missing proteins in each category

Category: Complete
KOG0018, KOG0025, KOG0271, KOG0285, KOG0292, KOG0357, KOG0363, KOG0434, KOG0563, KOG1211, KOG1241, KOG1942, KOG2311, KOG2606, KOG2623, KOG2638 KOG2707, KOG2785, KOG2967, KOG3405

Category: Partial
KOG0025, KOG0292, KOG0357, KOG0434, KOG0563, KOG1211, KOG1241, KOG1942 KOG2311, KOG2606, KOG2623, KOG2707, KOG3405
