# Supplementary material for: Transcriptome profiling of the dynamic life cycle of the scypohozoan jellyfish Aurelia aurita
Source: BMC Genomics. 2015 Feb 14;16(1):74. doi: 10.1186/s12864-015-1320-z (PMC4334923; doi:10.1186/s12864-015-1320-z)
Supplement: Additional file 9: — Comparison of the Aurelia Roscoff CL390 deduced protein sequence with that of the Aurelia RS strain. The putative signal peptide sequence is underlined, arginine repeats are highlighted in grey, tryptophan residues are highlighted in yellow, and the seven amino acids shown to induce strobilation in the Roscoff strain are marked in red. The sequence of CL390-like was found in a misassembled contig and therefore it was isolated by PCR from strobila RNA and sequenced. The CL390-like nucleotide sequence can be found in the GenBank under the accession number KM587721. [file 12864_2015_1320_MOESM9_ESM.pdf]

```
RedSea  CL390  MKTLSILLLIVLSVVLCAATREAVEETAKDALRPAEDTIQPVETDSYEPEEDQAESEEEP 60
Roscoff CL390  MKVLSILILIGLSVFSCAATKDAEEKITKDAIRTAGDTIEPVESDSDEPEQDQDESEEEP 60
               **.****:** ***. ****::* *: :***:*.* ***:***:** ***:** ******

RedSea  CL390  TEAESTNDDQDEENSAGTIKVSSDSFFWRRRRRSTRRRCYFARRRYFARRRYIARRRYTA 120
Roscoff CL390  NEAESTSDDQDEESSA-TTEASSDPIFWRRR--STRRRCYYARRRYIARRRYIARRRCYR 117
               .*****.******.** * :.***.:*****  *******:*****:**********

RedSea  CL390  RRGPIAHRRRRGVILLRRRSSLRRRRSSLRRRSGGRRRSSVRITTIR-WSSRRRG      173
Roscoff CL390  RR---SFRRR---LSFRRRWSRRR---WLRRRFSYRRRWSRRRSLRRRFSFRRRG      163
               **   :.***   : :*** * **    **** . *** * * :  * :* ****
```
